# Differential Flight Responses of Sympatric Raptor Species to Weather Conditions and Extreme Temperature Events

**DOI:** 10.1002/ece3.70658

**Published:** 2025-02-19

**Authors:** Lara Naves‐Alegre, Hernán García‐Mayoral, Jon Morant, Juan Manuel Pérez‐García, Andreia Dias, Elvira Cano‐Montes, Ángel Sánchez, Víctor García‐Matarranz

**Affiliations:** ^1^ University Institute for Agro‐Food and Agro‐Environmental Research and Innovation (CIAGRO‐UMH) Universidad Miguel Hernández de Elche Elche Spain; ^2^ Independent Researcher; ^3^ Department of Ecology University of Alicante Alicante Spain; ^4^ Conservation Action Area, Spanish Ministry of Ecological Transition and the Demographic Challenge Madrid Spain; ^5^ Sociedad de Gestión Pública de Extremadura Badajoz Spain; ^6^ Servicio de Conservación de La Naturaleza y Áreas Protegidas Junta de Extremadura Mérida Spain

**Keywords:** behavioral flexibility, climate change, flight behavior, heatwaves, meteorology, raptors

## Abstract

Climate change has increased the frequency, severity, and duration of extreme weather events, for example, heatwaves, underscoring the need to comprehend their impact on animal behavior. Flying organisms, particularly birds, are greatly affected by changes in atmospheric conditions and may modify their speed or direction, adjust their flight strategy, and even make decisions on whether to fly based on weather. In this study, we assessed flight‐related parameters in three GPS‐tagged raptor species: the golden eagle ( *Aquila chrysaetos* ), the Bonelli's eagle (*Aquila fasciata*), and the Spanish eagle (*Aquila adalberti*), in relation to weather conditions and heatwaves. The results showed that the three species varied in their flight patterns despite similar environmental conditions, including temperature, precipitation, wind speed, and atmospheric pressure. Each species exhibited unique strategies and responsiveness to heatwaves, reflecting diverse adaptive capacities and behavioral flexibilities. Specifically, Bonelli's eagle displayed comparatively minor adjustments in its flight strategy during periods of extreme temperature, contrasting with the pronounced behavioral variations observed in the golden eagle. These findings suggest that extreme and unpredictable weather events, particularly heatwaves, may impact raptor species differently. An understanding of how extreme weather events may impact individual fitness, through modifications to energy expenditure and foraging practices, is essential for predicting their potential impact on long‐term population dynamics.

## Graphical abstract

Flying organisms, such as birds, alter their flight behavior in response to atmospheric conditions, and climate change has exacerbated extreme weather events like heatwaves, impacting their flight. This study examined the flight patterns of three raptor species (Golden eagle, Bonelli's eagle, and Spanish eagle) using GPS data and found that each species exhibited unique adaptations and responses to heatwaves and weather conditions. This research highlights the importance of understanding how extreme weather events affect energy expenditure and foraging behavior, which is crucial for predicting long‐term impacts on bird populations.

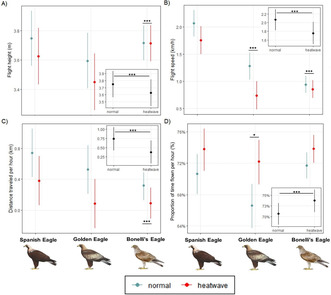

## 1 Introduction

Anthropogenic climate change is one of the greatest threats to biodiversity and wildlife conservation (Wiens 2016; Antonelli 2022). In recent decades, multiple biological responses to changes in overall climate conditions have been shown, including shifts in species distribution and their ecological niches, variations in population size, behavior, and finally, changes in fitness (du Plessis et al. 2012; Lemoine et al. 2013; Cunningham, Martin, and Hockey 2015; Ameca, Chamart, and Garber 2023; Vad et al. 2023). In addition to rising global temperatures, climate change has intensified the frequency, severity, and duration of extreme weather events worldwide (i.e., events that exceed typical pre‐existing conditions, e.g., floods, droughts, storms, heatwaves, and cold waves) (Hansen, Sato, and Ruedy 2012; Seneviratne et al. 2012; Perkins‐Kirkpatrick and Lewis 2020). Climate change is not having uniform effects across all regions. The Mediterranean region is predicted to be one of the most vulnerable area to climate change, with a higher temperature increase than the global average (Myers et al. 2000; Seneviratne et al. 2016), and prolonged and intense extreme events such as droughts (Pulquério et al. 2015) and heatwaves (Perkins‐Kirkpatrick and Lewis 2020).

Studies on the impact of climate change typically focus on long‐term changes; however extreme, weather events can lead to immediate and localized impacts (Kay et al. 2021; Naveda‐Rodríguez and Rush 2023). Heatwaves have been broadly defined as prolonged periods of unusually high temperatures compared to normal conditions for that specific region and season (Fragueira et al. 2021; Corregidor‐Castro et al. 2023). Although organisms have evolved to cope with predictable environmental variations, unexpected events can negatively impact survival, reproduction, habitat selection, and the resources on which they depend (Wingfield et al. 2017; Kreling et al. 2021; Naveda‐Rodríguez and Rush 2023; Almaraz and Green 2024). Nevertheless, different adaptative strategies in response to extremely high temperatures have been documented. In mobile organisms, behavioral responses include shifting activity patterns to cooler periods, reducing overall activity levels, or engaging in migrations (Møller 2011; Wingfield et al. 2017; Kreling et al. 2021; Ameca, Chamart, and Garber 2023). Some studies have also shown a negative effect of extreme temperatures on reproductive efficiency (Catry et al. 2015; Fragueira et al. 2021; Corregidor‐Castro et al. 2023). In addition, several cases of mortality have been documented during periods of extreme temperatures, causing an instantaneous demographic effect (Bailey and van de Pol 2016; Costa et al. 2023). However, the study of this type of immediate effect remains far less explored than the influence of long‐term climate change (Wingfield and Kitaysky 2002; Vázquez et al. 2017).

Thermal tolerance is a fundamental trait determining species' vulnerability to increasing temperatures. When temperatures exceed the tolerated thermal zone, endothermic organisms use behavioral and physiological strategies to lose heat and maintain a constant body temperature (Pörtner 2001; Catry et al. 2015). Behavioral flexibility allows these individuals to adapt to environmental changes, including extreme weather (van Buskirk, Candolin, and Wong 2012; Berger‐Tal et al. 2016; Beever et al. 2017). Behavioral reactions are more immediate than demographic or distribution changes, providing an early signal of the effects of climate stressors (Berger‐Tal et al. 2011, 2016). The immediate responses of animals to the weather and extreme conditions can have important consequences for the recovery and maintenance of populations and communities (Strandberg et al. 2010; Danner, Coomes, and Derryberry 2021; Kreling et al. 2021). Measuring these responses requires high‐resolution data (Lanzone et al. 2012). The advent of sophisticated telemetry devices has provided precise, near‐real‐time movement data, enabling the study of species' space use and energy expenditure (Hetem et al. 2012; Duriez et al. 2014; Wilson et al. 2020). This allows for a deeper understanding of behavioral changes related to organismal movement strategies.

Movement is a fundamental signature of animal ecology, being directly related to multiple processes, for example, energy expenditure, resource acquisition, and reproduction, which may ultimately affect fitness (Nathan et al. 2008; Kranstauber et al. 2011). Flying organisms are greatly affected by fluctuations in atmospheric and weather factors that support efficient flight (Mitchell et al. 2012; Duerr et al. 2015). Flight behavior in birds, including adjustments in speed, direction, and the decision to fly, is influenced by weather conditions (Duerr et al. 2015; Poessel et al. 2018; Panuccio, Mellone, and Agostini 2021). Moreover, the flight strategy (i.e., soaring, gliding, or flapping) also depends on the species' morphology and size (Ruxton and Houston 2004; Agostini, Panuccio, and Pasquaretta 2015; Williams et al. 2020), life history, and sociality (Beauchamp 2023). As a result, strong selection pressures are predicted for energy‐minimization strategies in response to weather, since survival and reproduction may depend on flying behavior (Lanzone et al. 2012). Among birds, raptors are apex predators that play a fundamental role in ecosystem functioning, making them a critical group for understanding how species cope with environmental change (Donázar et al. 2016). Eagles, in particular, are close to the maximum body size feasible for flying species, which, combined with their reliance on flight for essential behaviors such as hunting and migration, makes them highly sensitive to weather variations (Lanzone et al. 2012; Rus et al. 2017). Their dependence on specific weather conditions for efficient flight and thermoregulation heightens their vulnerability to extreme weather events, positioning them as valuable indicators of climate‐related impacts on wildlife. Although there are a few studies comparing flight variables among closely related raptor species, these mainly focus on migration patterns (Vidal‐Mateo et al. 2022; Chiatante et al. 2023). Research on how resident species respond to weather and extreme climate events remains limited.

In this study, for the first time, we analyzed the effect of varying weather conditions on flight behavior of three GPS‐tagged raptor species: the Spanish eagle (*Aquila adalberti*), the golden eagle (*Aquila chrysaetos*), and Bonelli's eagle (*Aquila fasciata*), inhabiting a Mediterranean area. We compared flight behaviors among territorial and resident individuals of these three species and their flexibility during extreme weather events (i.e., heatwaves) in a sympatric area. Specifically, we evaluated short‐term responses related to flight metrics and energy expenditure, including flight height, speed, distance traveled per hour, and time spent flying, using detailed high‐frequency GPS data. We hypothesize that the three species will exhibit different responses to weather variables (i.e., temperature, precipitation, atmospheric pressure, and wind speed) due to their distinct morphological (e.g., body size and wing span) and ecological traits (e.g., biogeographic distribution and diet) (Lanzone et al. 2012; Poessel et al. 2018; Cheney et al. 2021). Specifically, we predict that the golden and Spanish eagles will respond similarly to weather variations compared to Bonelli's eagle. The golden eagle (from 2.9 to 6 kg; Arroyo 2004) and Spanish eagle (2.5–4.8 kg; Mariano González 2016) are expected to show faster flight speeds, longer distances traveled, and increased flight time at higher temperatures and stronger winds (Black and Borowske 2009; Agostini, Panuccio, and Pasquaretta 2015). In contrast, Bonelli's eagle, being smaller (1.6–2.5 kg; Ontiveros 2016) and having greater maneuverability, may be more sensitive to wind intensity, affecting its flight behavior and reducing time spent flying. Additionally, we predict that the flight behavior of Bonelli's eagle will be affected to a lesser extent than the other two species by precipitation as it is adapted to tropical and subtropical regions of Asia (Ferguson‐Lees and Christie 2001). We also hypothesize that behavioral responses to unpredictable extreme weather events, like heatwaves, will differ among species due to their biogeographical backgrounds. We predict that the golden eagle will exhibit more pronounced changes, leading to increased energy expenditure (Wingfield et al. 2017), while Bonelli's and Spanish eagles, being more adapted to warm conditions, may show less intense or no behavioral responses (Gil‐Sánchez et al. 2004; Fernández et al. 2009; López‐Peinado and López‐López 2024; Moleón et al. 2024). Overall, our results will help determine the immediate behavioral responses of these raptors to increasing heatwave intensity and frequency in Mediterranean areas.

## 2 Material and Methods

### 2.1 Study Area

The study was conducted in the regions of Extremadura and a small portion of Castilla La Mancha, southwestern Spain, covering a total of 33,173 km^2^. Both are a biodiversity hotspot (Mittermeier et al. 2011), with a Mediterranean climate, semiarid to dry subhumid, with mild temperatures during the winter and rainfall concentrated in the fall (Felicísimo Pérez 2001).

### 2.2 Data Collection

We used data from 7 golden eagles, 18 Bonelli's eagles, and 7 Spanish eagles, equipped with solar‐powered GPS/GSM transmitters (Model OT‐50; Ornitela https://www.ornitela.com/) from 2021 to 2024 (see Table S1 for details about the number of tracking days per individual). Eagles were captured using radio‐controlled bow‐net traps and transmitters were deployed as backpacks by a Teflon harness with a central ventral rupture point (see details in García, Iglesias‐Lebrija, and Moreno‐Opo 2021). All tracked individuals were territorial adults; however, their sex was unknown as no sexing analysis was conducted. The combined weight of the tagging equipment, including the transmitter (50 g), harness, and rings, represented less than 3% of the total body weight, which was established as a threshold to avoid any negative effects on the birds (Sergio et al. 2015; Bodey et al. 2018). All transmitters included a triaxial accelerometer datalogger which measures the instantaneous acceleration (Graf et al. 2015).

### 2.3 Data Processing

We initially obtained 5,378,944 raw GPS positions. Since the GPS devices were configured to record positions at different frequencies (mean ± SD: 1.55 ± 3.5 min) from 1 h before sunrise to 1 h after sunset, data were standardized by resampling to 5‐min intervals (i.e., the shortest interval common to all individuals) with a 15‐s tolerance, including only positions geolocated with four or more satellites. In addition, we eliminated spatial outliers related to false positions by defining the most extreme interquartile range (IQR) of latitude and longitude values as IQR=Q95−Q5; with the upper limit of typical values being Q95+IQR and the lower limit of typical latitude and longitude values being Q5−IQR, where Q5 is the quantile that includes 5% in the lower tail of the data distribution and Q95 is the quantile includes 95% of the data. These steps resulted in a final dataset of 1,048,575 valid positions. To accurately calculate flight‐specific metrics, for example, *distance traveled per hour* and the *proportion of time spent in flight per hour*, we classified each location as corresponding to a *flying* or *nonflying* bird. To do this, we first modeled *flight speed* as the sum of two beta‐binomial distributions, that is, a beta‐binomial function for *flying* data and another for *nonflying* data, which enabled us to classify 98% of the data. We selected beta‐binomial distribution due to its flexibility in modeling overdispersed data, allowing us to capture the inherent variability in *flight speeds* as well as uncertainty in the speed measurements. Next, we trained the k‐nearest neighbors (k‐NN) machine learning model using these previously categorized data, along with the accelerometer module (i.e., *acc*) and the accelerometer y‐axis (i.e., *acc‐y*), which allowed us to categorize them as *flying* (*n* = 249,137) or *nonflying* data (*n* = 799,438) those that were initially uncertain (2% of the data). To accurately distinguish passive soaring flight, which may have low acceleration similar to *nonflying* data, we combined *flight speed* data with accelerometer readings to ensure proper identification of soaring behavior and avoid misclassification (see Appendix S1 for further details). We also assumed that within the short 5‐min time intervals, the same flight category persisted, allowing us to calculate the total time an individual spent *flying* versus *nonflying* over the total recorded period.

We used year‐round data, so we established a categorical variable for the *breeding season* to account for variations in movement patterns and energy expenditure patterns of the three species considered during and outside of this season (Fernández et al. 2009; Pérez‐García et al. 2013; Poessel et al. 2016). We divided the data into two periods: *breeding season* (incubation, breeding, and chick dependency) and *nonbreeding season*, specific to each species (see Table S2 for exact dates). We do not use only the data belonging to the breeding season (centered on the summer months, where heatwaves predominate) because the *nonbreeding season* of the Bonelli's eagle begins in August and this would cause us to exclude a large number of important data. Moreover, the reproductive success of each individual for each year was not considered since these data were not available for all of them. Data were processed and cleaned using Python (Python 2021).

### 2.4 Flight Behavior Variables

Initially, we extracted the *instantaneous speed* (also called *ground speed*) and *flight height* variables provided by the transmitter. We grouped position‐level data into 1‐hour intervals to calculate flight‐related variables that require multiple standardized data points, for example, the *proportion of time flown*. Since these variables depend on the number of positions per unit of time, we standardized them according to the number of positions recorded per individual per hour, accounting for occasional missing data (e.g., due to GPS errors; Silva et al. 2017). The hourly scale was chosen as it is ideal for analyzing the influence of meteorological factors on short‐term flight behavior, whereas longer time scales (e.g., daily data) would not capture rapid behavioral responses (McClintock et al. 2014). This approach reduced the dataset to 163,406 hourly records that were used for the analyses: 37,587 for Spanish, 45,698 for golden, and 80,121 for Bonelli's eagle (Table S1). We defined the variables *flight speed* (km/h), i.e., mean *instantaneous speed* measured by the transmitter per hourly data, considering all measurements taken during that hour; and *flight height* (m), average height above the ground at which the bird flies per hour, calculated by subtracting the terrain elevation (obtained from NASA SRTM https://www.earthdata.nasa.gov/ digital elevation 30 m with a resolution of 30 × 30 m) from the altitude above sea level (m; measurement provided by the GPS transmitter) (see Péron et al. 2020 for details). We defined *distance traveled per hour* (km) by calculating the sums of Euclidean distances in the X‐axis between all the instant locations registered per hour, which provides information on the degree of mobility. We also used *proportion of time flown per hour* as a proxy of the energetic expenditure (Hills, Mokhtar, and Byrne 2014; Wilson et al. 2020; Morant et al. 2022). Although *flight speed* and the *distance traveled per hour* may seem directly related, they actually represent different aspects of flight behavior. Mean flight speed provides insight into the bird's velocity during active movement periods, while the distance traveled per hour reflects the total ground covered in that timeframe. A bird could maintain a relatively high speed but not cover much distance if it performs circling or hovering maneuvers, indicating distinct behavioral patterns that are not captured by speed alone.

### 2.5 Weather Variables

Meteorological data were extracted from the website https://www.worldweatheronline.com/, which provides real (not modeled) weather information at hourly and ground level from the nearest meteorological station to each fix (Terence et al. 2015). We specifically selected the variables whose effect had been previously tested in the movement of raptors, that is, *temperature* (°C), *precipitation* (mm), *relative humidity* (%), *wind speed* (km/h), and *atmospheric pressure* (hPa) (Walls, Kenward, and Holloway 2005; Vansteelant et al. 2014). Furthermore, Pearson correlations between these meteorological variables were calculated to select the most appropriate ones to include in the models. Those variables that were highly correlated (*r* > |0.5|) were discarded and retained only those that made the most biological sense, that is, *temperature*, *precipitation*, *wind speed*, and *atmospheric pressure* (Figure S6) (Rus et al. 2017). Finally, we identified the variable *heatwave* as a period of 2 or more consecutive days with more than 37°C (Catry et al. 2015; Corregidor‐Castro et al. 2023). Thus, all hourly data included within days above 37°C were categorized as “heatwave” (*n* = 17,669), and those lower temperatures were categorized as “normal” (*n* = 145,737).

### 2.6 Statistical Analyses

We used generalized linear mixed models (GLMM) to analyze the influence of weather variables and heatwaves on the flight of the three study species. For this purpose, we utilized as response variables referring to flight metrics: (i) *flight height*, (ii) *flight speed*, (iii) *distance traveled per hour*, and (iv) *proportion of time flown per hour*. We built separate GLMMs for each flight metric and each weather factor, that is, *temperature*, *precipitation*, *atmospheric pressure*, and *wind speed*, and an additional model to analyze the effect of *heatwaves*. For each model, we included the interaction between the weather variable (or *heatwave*) and *species* to assess whether the flight behavior of different species responded differently to changes in weather conditions or extreme weather events. In addition, we included in all models the *hour* of the day and the *breeding season*, as these are variables that have been shown to have a great influence on the flight patterns of birds (Morant et al. 2023). All weather variables included in the models were scaled due to the differences in magnitude orders. The *individual identity* was included as a random factor. We used the *glmer* function in “lme4” package for GLMM analyses (Bates et al. 2007) in R 3.5.1 (R Core Team 2022).

## 3 Results

**FIGURE 1:**
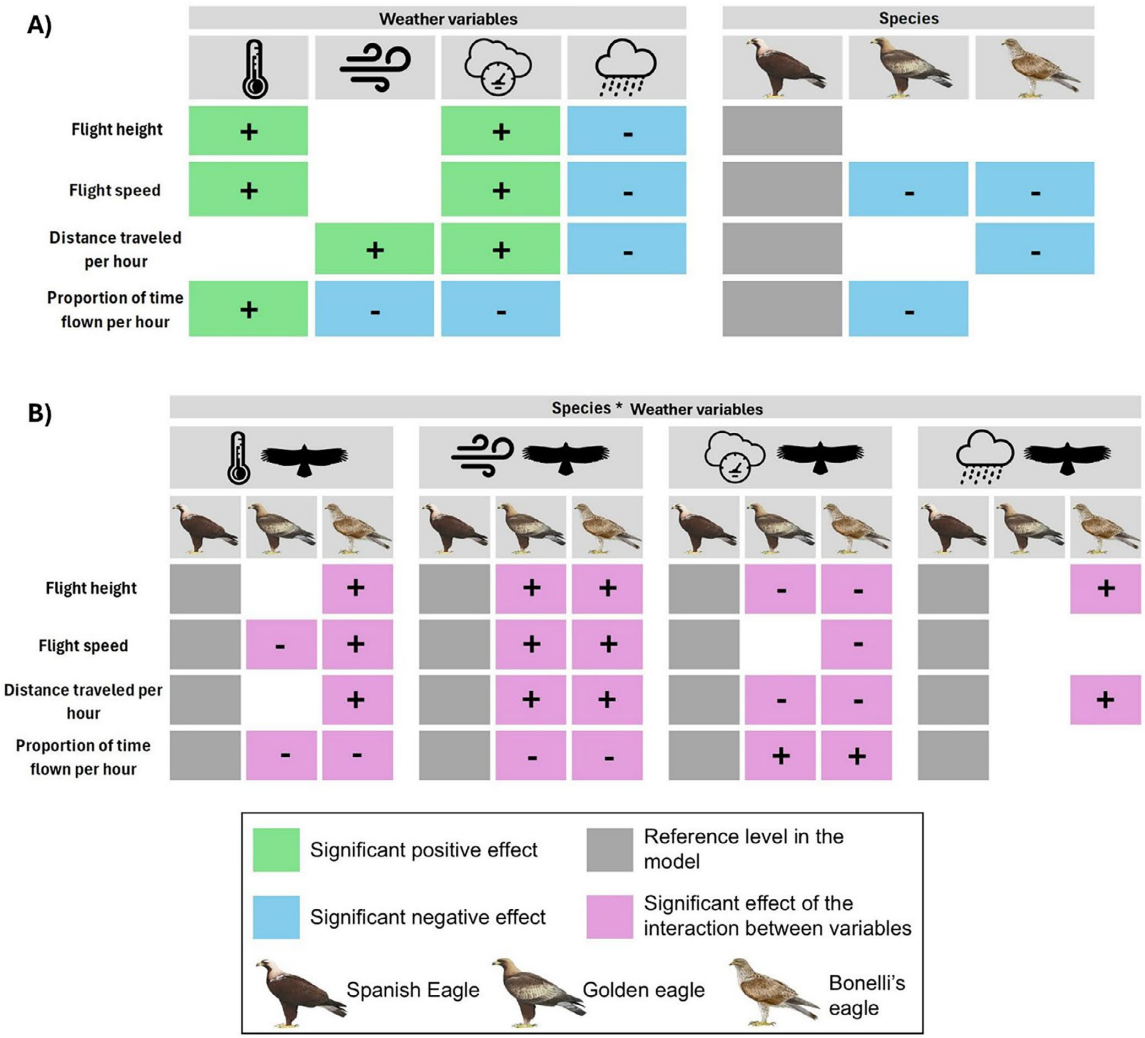
Results obtained from GLMMs show (A) the significant effects of weather variables (i.e., temperature, wind speed, atmospheric pressure, and precipitation, represented in that order) and the differences between species in the different variables related to flight strategy. (B) The significant results of the interactions between weather variables and the species. For more details on the results of the interactions between variables, see Appendix 1. Raptor illustrations reproduced from: www.birdsoftheworld.org.

The results showed differences in flight variables, that is, *flight height* and *speed*, *distance traveled per hour*, and *proportion of time flown per hour*, among the three raptor species, although the standard deviations are very high due to the large variation between individuals (Table 1). On the one hand, GLMM results indicate differences in the different flight variables between the species. The golden and Bonelli's eagles flew significantly slower than the Spanish eagle. The golden eagle, moreover, spends a lower *proportion of time flown per hour* than the Spanish eagle. On the other hand, we also found that Bonelli's eagle flew significantly shorter *distances per hour* than the Spanish eagle (Figure 1A).

**TABLE 1:** Means and standard deviations of all variables related to flight strategy and energy expenditure by the three raptor species studied.

	Spanish eagle	Golden eagle	Bonelli's eagle
Flight height (m)	117.97 ± 182.75	90.62 ± 162.90	107.37 ± 197.06
Flight speed (km/h)	16.75 ± 21.45	14.43 ± 20.98	13.10 ± 19.26
Distance traveled per hour (km)	7.94 ± 10.22	7.14 ± 10.21	5.50 ± 8.16
Proportion of time flown per hour (%)	71.57 ± 33.29	67.77 ± 32.84	70.77 ± 33.08

Analyses also showed how weather variables differentially influenced flight‐related variables (Figure 1A). Temperature significantly influenced most flight‐related variables, having a positive effect on *flight height* and *speed* and on the *proportion of time flown per hour*. Similarly, wind speed also had an influence on flight patterns, positively affecting *distance traveled per hour*, and having a negative effect on the *proportion of time flown per hour*. Atmospheric pressure had a significant and positive impact on *flight height* and *speed*, and *distance traveled per hour*. However, it had a significant negative effect on the *proportion of time on flight*. Also, precipitation negatively influenced *flight height* and *speed*, and *distance traveled per hour*, with no influence on the *proportion of time flown per hour* (Figure 1A, Tables S3–S6).

The results showed that the three species showed differential responses to weather conditions (Figure 1B). Bonelli's eagles exhibited a stronger response to temperature compared to golden eagles and Spanish eagles in terms of *flight height*, *flight speed*, and *distance traveled per hour*. These three variables exhibited a more pronounced change with rising temperatures. Regarding atmospheric pressure, Bonelli's eagles had a weaker response in *flight speed* compared to the other two species. When considering *precipitation*, this species showed a significantly less pronounced decrease in both *flight height* and *distance traveled per hour*. Additionally, both Bonelli's and golden eagles responded less to temperature in the *proportion of time flown per hour* than Spanish eagles, with a comparatively smaller increase in this variable. In terms of wind, Bonelli's and golden eagles exhibited divergent responses from Spanish eagles across all flight metrics. Both species also responded differently to atmospheric pressure in *flight height*, *distance traveled per hour*, and *proportion of time flown per hour*. Furthermore, the increase in *flight speed* observed in golden eagles in response to rising temperatures was less pronounced in comparison to both Spanish and Bonelli's eagles.

**FIGURE 2:**
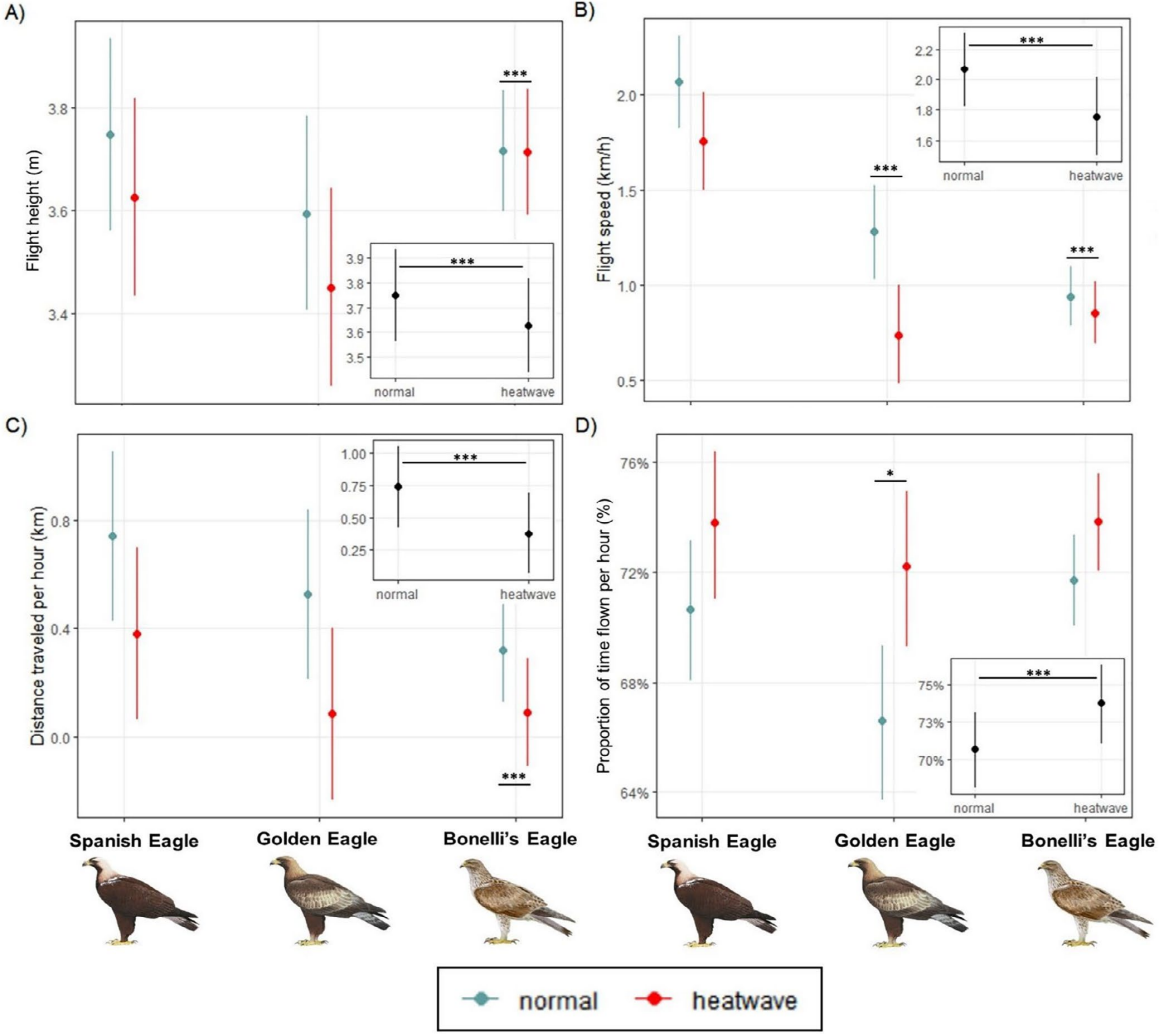
Plots of the models showing the effect of heatwaves on flight height, flight speed, distance traveled per hour, and proportion of time flown per hour for Spanish, golden, and Bonelli's eagles. The smaller inset boxes represent the overall influence of heatwaves on each flight variable, indicating the significant effects (*p* < 0.05). Large plots show variations in the flight variables during heatwaves compared to normal periods (i.e., normal) for the four species, indicating the significance level of the interaction between the species and heatwave. See Appendix 1: Table S7 for further details. Some of the variables (i.e., flight height, speed, and distance traveled per hour) were transformed for the analysis (see material and methods section) and are plotted on a logarithmic scale on the vertical axis. Significance levels: *p < 0.05 and ***p < 0.001. Raptor illustrations reproduced from: www.birdsoftheworld.org.

The analyses showed that *heatwaves* exerted a significant negative influence on *flight height* and *flight speed*, and on the *distance traveled per hour* for all the species. However, models revealed a contrasting positive effect on the *proportion of time in flight per hour* for the three species (Figure 2). Furthermore, the interactions between *species* and *heatwave* were found to be statistically significant by species (Figure 2). Specifically, the Bonelli's eagle showed significantly less variation in *flight height* and *speed*, as well as in the *distance traveled per hour* during heatwaves in comparison with the Spanish and golden eagles. In contrast, the golden eagle exhibited greater changes in its *flight speed* and the *proportion of time flown per hour* during heatwaves, in comparison with the Spanish eagle (Figure 2, see Appendix S1: Table S7 for further details).

Finally, our findings revealed that both the covariates of hour and breeding season significantly influenced all models. The time of day had a positive effect on all analyzed flight characteristics, except for the *proportion of time flown per hour*, for which it had a negative effect. Conversely, being outside the breeding season resulted in a negative impact on all flight‐related variables, except for the *proportion of time flown per hour*, which significantly increased during this period of the year (see Appendix S1: Tables S3–S7).

## 4 Discussion

Our results showed differential responses in flight behavior to extreme weather events in three raptor species inhabiting a Mediterranean area of the Iberian Peninsula. We found that the three species differed in their flight patterns under similar environmental conditions, which may be the result of their distinct morphological (e.g., body size) and behavioral traits (e.g., hunting methods). Notably, extreme temperatures, particularly heatwaves, had a clear impact on their flight behavior. However, the three species exhibited different strategies and varying intensities in their responses to these extreme events, highlighting the greater responsiveness of the golden eagle. This variation in behavioral responses may reflect the differential impact of extreme climatic events among species, as well as their distinct adaptive capacities (i.e., behavioral flexibility) shaped by their historical biogeographic ranges and morphological traits.

According to our first hypothesis, we found that raptors can adapt their flight behavior to weather conditions in a short‐term time scale, albeit with interspecific variation. Other studies have also shown how movement patterns vary among avian species depending on changes in weather conditions (Shamoun‐Baranes, Liechti, and Vansteelant 2017; Poessel et al. 2018). However, we also found previous research in which the influence of most of the meteorological variables had no significant effect on flight characteristics, assuming that other factors may play a more important role (e.g., availability of resources or inter‐ and intraspecific interactions) (Arrington 2003; DeVault et al. 2005).

Air temperature is a factor that has been found to greatly influence animal behavior across different taxa (e.g., O'Connor, Gilbert, and Brown 2011; Briffa, Bridger, and Biro 2013; Lemoine et al. 2013; Woodroffe, Groom, and McNutt 2017; Rafiq et al. 2023). Our results show that temperature positively affected raptor flight height and speed and proportion of time flown per hour. Consistent with these findings, previous studies have shown that in general warm temperatures favored flight and hunting behaviors more than other weather variables, decreasing energy expenditure (Hiraldo and Donazar 1990; DeVault et al. 2005). Moreover, we did not find a general effect of temperature on the distance traveled by birds. This differs from what has been found in other research, where the migration time and distance traveled during dispersal were influenced by temperature (Walls, Kenward, and Holloway 2005; Duerr et al. 2015; McCaslin, Caughlin, and Heath 2020). Partially aligning with our prediction, we observed that the golden and Spanish eagles responded similarly to temperature variations in terms of flight altitude and distance traveled per hour. This result may be explained by the role of temperature in generating thermal currents, which can facilitate more energy‐efficient flights for large birds such as these species (Kerlinger and Moore 1989; Poessel et al. 2018). However, our results also showed that the golden eagle was less influenced by increasing temperature compared to the Spanish eagle in the proportion of time flown per hour and flight speed. Although the differences in response between the Spanish and golden eagles were small, they may be related to the fact that soaring flight in golden eagles is also influenced by other factors, for example, gravity waves, and not just by thermal currents (Carrard et al. 2024).

Wind speed has been described as a fundamental factor in the movement of flying animals (Shamoun‐Baranes et al. 2007; Vansteelant et al. 2014; Harel et al. 2016). Accordingly, our analysis showed that wind speed did indeed have an overall effect on flight variables directly related to energy expenditure, such as distance traveled per hour and proportion of time flown per hour. However, we did not find an effect of wind on flight height, as has been evidenced in other work (Lanzone et al. 2012; Poessel et al. 2018). The latter may be explained by the fact that we did not take wind direction into account in our analyses, given that our objective did not involve the analysis of energy expenditure or changes in flight strategies. In line with our prediction, we also found clear differences between species in flight characteristics depending on wind speed (Shamoun‐Baranes et al. 2007). These differences may stem from the direct influence of specific morphological traits (e.g., tail length) on flight efficiency in response to wind conditions (Black and Borowske 2009; Limiñana et al. 2013; Cheney et al. 2021). In this way, and according to our predictions, the Bonelli's eagle reduces the proportion of time flown per hour to a greater extent when wind strength increases, which may also be related to its smaller body size and lower capacity to respond to strong winds, in comparison with the other two species. However, contrary to our initial expectations based on the species' body size, we found that the Spanish and Golden eagles respond differently to variations in wind conditions. In contrast, the Golden eagle and Bonelli's eagle exhibit more similar responses. These differences may be explained by the distinct hunting strategies of the species. The Spanish eagle primarily relies on a sit‐and‐wait strategy, spending considerable time perched in ambush and hunting in specific, localized areas (Nadjafzadeh, Hofer, and Krone 2016). The Golden eagle, by contrast, engages in long flights, covering large areas to search for potential prey (Collopy 1983). The Bonelli's eagle, like the Golden eagle, employs a more active pursuit strategy, hunting while flying and using direct flight paths to capture prey (Martínez et al. 2014). Consequently, flight characteristics of Golden and Bonelli's eagles, which are more reliant on active searching and pursuit, may be more affected by wind conditions, while the Spanish eagle, which spends more time perched, may be less influenced.

Atmospheric pressure had a generalized positive effect on flight height, flight speed, and distance traveled, consistent with findings from previous studies where it has been shown that pressure is positively related to the development of Type I thermal air currents (i.e., rising column of warm air generated by uneven heating of the Earth's surface) (Kerlinger and Moore 1989; Duerr et al. 2015). In contrast, precipitation had a negative effect on flight height and speed, and distance traveled per hour. These results differ from those found during migration, in which precipitation had no general significant effect, although it did have a differential effect depending on the ecoregion (Duerr et al. 2015). Migration is a time‐sensitive process, and thus, migrating individuals may be less prone to modify their behavior in response to adverse environmental conditions, as migration represents a crucial phase in the life cycle of many species (Åkesson et al. 2017). Additionally, we found that Bonelli's eagle showed less alteration in flight height and distance traveled per hour in response to increasing temperatures compared to Spanish and golden eagles. This finding aligns with our initial prediction, based on the fact that the distribution range of this species primarily encompasses tropical and subtropical regions (Ferguson‐Lees and Christie 2001).

Overall, our results indicated that the Bonelli's eagle exhibited the most distinct flight behaviors in response to weather conditions compared to the Spanish and golden eagles, especially regarding changes in temperature and precipitation. These findings are consistent with our initial expectations and are likely influenced by Bonelli's eagle's different morphology. Compared to the Spanish and golden eagles, Bonelli's eagle has a smaller body size and shorter, broader wings relative to its body, which provide greater maneuverability but reduced lift (Gil‐Sánchez, Molino, and Valenzuela 1996; Gil‐Sánchez et al. 2004; Ontiveros and Salvador Milla 2016; Moleón et al. 2024). These morphological differences are related to variations in behavioral patterns, particularly regarding their primary foraging techniques. The Bonelli's eagle primarily preys on rabbits (*Oryctolagus cuniculus*), pigeons (*Columba sp*.), and similar‐sized birds (Gil‐Sánchez et al. 2000; Moleón et al. 2009), while the Spanish eagle not only is specialist hunter of rabbits but also scavenges frequently (Sánchez et al. 2008; Margalida et al. 2017). In contrast, the golden eagle is the most generalist of the three eagles, with a diet that adapts to prey availability in the area (e.g., rabbits, hares (*Lepus sp*.), and pigeons), and that also includes important quantities of carrion (Sánchez‐Zapata et al. 2010; Clouet et al. 2017).

Extreme heat significantly impacts short‐term animal behavioral patterns (Hetem et al. 2012; Levy et al. 2016; Funghi et al. 2019). In response to higher average heat stress, our results reveal a shift in behavioral patterns, with individuals spending more time in flight during heatwave conditions compared to normal temperature days. This behavior likely serves as a mechanism for heat dissipation, considering that temperatures at flight heights may be slightly lower than at ground level (Linacre 1982; du Plessis et al. 2012), although with higher energy costs (Morant et al. 2023). Behavioral shifts during heatwaves diverged from general responses to temperature, except for the proportion of time spent flying, which increased despite reduced travel distance. This phenomenon may be influencing foraging patterns, aligning with previous studies demonstrating similar impacts of elevated temperatures on animal behavior, underscoring the fitness costs of such changes (du Plessis et al. 2012; Funghi et al. 2019; Cunningham, Gardner, and Martin 2021; Fragueira et al. 2021). As expected, Bonelli's eagle was the species that modified its flight behavior the least during heatwaves, possibly reflecting a lower energy investment under these conditions. This may be due to its adaptation to warmer climates, with temperature being the factor that most influence its reproductive distribution (Solanou et al. 2022; López‐Peinado and López‐López 2024), or its smaller body size, which may facilitate temperature regulation (Angilletta 2009; Gardner et al. 2011; Sejian et al. 2018). Bonelli's eagle was the smallest of the species studied, followed by the Spanish eagle, and finally, the golden eagle (del Hoyo, Elliott, and Sargatal 1994), potentially offering an adaptive advantage in extreme heat. As predicted, the golden eagle exhibited the greatest behavioral adjustments during extreme temperature events. This species has the widest range in the northern hemisphere among the studied eagles, spanning climates from Arctic tundra to moderately warm areas (IUCN 2022), making it the least adapted to the Mediterranean climate of this study site. This may suggest that this species presents higher behavioral flexibility as an adaptive strategy to thermal extremes (Jiguet et al. 2006; Jiguet, Brotons, and Devictor 2011) due to its lower capacity to dissipate heat given its larger size, leading to a higher energy expenditure (Wingfield et al. 2017), which may ultimately influence its population dynamics (Beever et al. 2017).

### 4.1 Limitations and Further Research

We should acknowledge several potential limitations. First, the differences observed between species in our results, as discussed in the general discussion, could be attributed to individual variation and the small sample sizes for the Spanish and golden eagle. Second, this study did not account for the sex of the individuals, a factor known to influence the movement patterns in some raptor species (García‐Jiménez, Pérez‐García, and Margalida 2018), although no sex‐based differences have been observed in others (e.g., Bonelli's eagle; Pérez‐García et al. 2013; Morollón, Urios, and López‐López 2022). Finally, the lack of data on successful reproduction may have influenced the observed responses to weather fluctuations and extreme heat events, not to mention that reproductive success itself may be affected by heatwaves (Marcelino et al. 2020).

This study adopts a behavioral approach to analyze immediate responses to environmental changes on an hourly scale. Understanding the effects of weather conditions on bird activity and flight patterns is crucial for predicting potential population changes in response to climate change models (Jiguet, Brotons, and Devictor 2011). However, future research should use data at longer time scales (e.g., daily or weekly data) to provide a more comprehensive view of how weather events influence thermal updrafts, energy expenditure, and foraging behaviors across species, given that heatwaves have been shown to impact foraging efficiency (e.g., home range), site selection, and parental attendance (Oswald et al. 2008; du Plessis et al. 2012; Cunningham, Martin, and Hockey 2015). Future studies should also consider reproductive success (e.g., whether the individual breeds in a given year or the number of surviving chicks) in addition to categorizing data as within or outside the breeding season, as discussed earlier. Moreover, examining whether these behavioral shifts produce cascading effects within the ecosystem is essential, particularly given the role of raptors as apex predators (Donázar et al. 2016). For instance, changes in foraging patterns may in turn influence population dynamics of key prey species (Nachappa et al. 2011). Identifying the traits that confer resistance or tolerance to climate change in various species is also vital for enhancing risk assessments and developing adaptation plans (Jiguet, Brotons, and Devictor 2011). Finally, these findings highlight the importance of understanding in‐flight behavioral variations in assessing wind energy hazards under future climate conditions, suggesting avenues for further research.

## Author Contributions

**Lara Naves‐Alegre:** conceptualization (equal), formal analysis (equal), funding acquisition (equal), investigation (equal), methodology (equal), software (equal), validation (lead), visualization (lead), writing – original draft (lead). **Hernán García‐Mayoral:** data curation (lead), methodology (equal), software (equal), writing – review and editing (equal). **Jon Morant:** conceptualization (equal), writing – review and editing (equal). **Juan Manuel Pérez‐García:** conceptualization (equal), validation (equal), writing – review and editing (lead). **Andreia Dias:** data curation (equal), investigation (equal), methodology (supporting), project administration (equal), writing – review and editing (equal). **Elvira Cano‐Montes:** data curation (equal), investigation (equal), writing – review and editing (equal). **Ángel Sánchez:** funding acquisition (equal), investigation (equal), project administration (equal), writing – review and editing (equal). **Víctor García‐Matarranz:** conceptualization (equal), funding acquisition (equal), investigation (equal), project administration (equal), supervision (equal), writing – review and editing (equal).

## Conflicts of Interest

The authors declare no conflicts of interest.

## Supporting information


Appendix S1.

## Data Availability

All data and code to replicate this study are available in a Figshare repository: https://doi.org/10.6084/m9.figshare.26431303.v1.
