# Supplementary material for: Differential Flight Responses of Sympatric Raptor Species to Weather Conditions and Extreme Temperature Events
Source: Ecol Evol. 2025 Feb 19;15(2):e70658. doi: 10.1002/ece3.70658 (PMC11836895; doi:10.1002/ece3.70658)
Supplement: Supplementary file 1 — Appendix S1. [file ECE3-15-e70658-s001.docx]

**Appendix S1**

**Differential flight responses of sympatric raptor species to weather conditions and extreme temperature events**

# Lara Naves-Alegre*, Hernán García-Mayoral, Jon Moran, Juan Manuel Pérez-García, Andreia Dias, Elvira Cano-Montes, Ángel Sánchez, Víctor García-Matarranz

# SECTION S1: Material and methods

**Table S1.** Summary of hourly ranges (i.e., hourly-data) per individual of each species, as well as the date of marking, the date of the last position and the total day with locations.

| **Species** | **Individual** | **Number of hourly-data** | **Tagging date** | **Last date** | **Days with locations** |
| --- | --- | --- | --- | --- | --- |
| Spanish imperial eagle (*Aquila adalberti*) | Azagala | 2753 | 02/08/2023 | 17/01/2024 | 168 |
|  | Calizo | 4035 | 01/01/2023 | 01/09/2023 | 243 |
|  | Imperial-181672 | 2239 | 25/01/2023 | 11/11/2023 | 290 |
|  | Imperial-1N | 6267 | 01/01/2023 | 27/12/2023 | 360 |
|  | Imperial-3X | 887 | 29/01/2023 | 30/08/2023 | 213 |
|  | Navilla | 17348 | 17/06/2021 | 30/10/2023 | 865 |
|  | Villamiel | 4058 | 08/01/2023 | 09/10/2023 | 274 |
|  |  |  |  |  |  |
| Golden eagle (*Aquila chrysaetos*) | Cansina | 6772 | 10/02/2023 | 15/02/2024 | 370 |
|  | Conquista | 11282 | 12/07/2020 | 11/11/2021 | 487 |
|  | Dolores | 1729 | 06/07/2023 | 19/09/2023 | 75 |
|  | Gato | 16840 | 29/09/2021 | 15/02/2024 | 869 |
|  | Guara | 8302 | 25/01/2023 | 16/02/2024 | 387 |
|  | Manchon | 327 | 31/01/2024 | 16/02/2024 | 16 |
|  | Servan | 446 | 25/01/2024 | 16/02/2024 | 22 |
|  |  |  |  |  |  |
| Bonelli's eagle (*Aquila fasciata*) | Africa | 3345 | 27/04/2023 | 23/01/2024 | 271 |
|  | Alange | 7570 | 16/12/2022 | 14/02/2024 | 425 |
|  | Caña | 1992 | 24/10/2023 | 16/02/2024 | 115 |
|  | Deleitosa | 8685 | 18/06/2021 | 20/11/2022 | 520 |
|  | Guadiana | 6456 | 24/12/2022 | 03/12/2023 | 344 |
|  | Latosa | 3992 | 13/06/2023 | 15/02/2024 | 247 |
|  | Latoso | 289 | 30/10/2023 | 15/02/2024 | 108 |
|  | Luca | 2567 | 11/01/2023 | 05/10/2023 | 267 |
|  | Mora | 1405 | 15/11/2023 | 16/02/2024 | 93 |
|  | Moro | 479 | 16/11/2023 | 14/02/2024 | 90 |
|  | Naturaleza | 1830 | 13/01/2023 | 30/08/2023 | 229 |
|  | Quinto | 534 | 10/01/2024 | 15/02/2024 | 36 |
|  | Retuerto | 7941 | 26/04/2022 | 22/07/2023 | 452 |
|  | Rubio | 6873 | 22/12/2022 | 15/02/2024 | 420 |
|  | Sereno | 324 | 20/12/2023 | 15/02/2024 | 57 |
|  | Torilla | 6425 | 07/01/2023 | 06/02/2024 | 395 |
|  | Torillo | 789 | 19/10/2023 | 14/02/2024 | 118 |
|  | Zorita | 18625 | 16/07/2020 | 06/02/2024 | 1300 |

**Determining whether the bird is flying or not.** We constructed the histogram of the flight speed data by grouping the speed values into integers (Figure S1). This allows us to distinguish two distinct groups of data, the data when the individual is not flying (i.e., perched), in which the velocity is zero, except for the error that may be present in the data collection, and the flight zone with velocities around 20-70 km/h. We fit the histogram values to the sum of two betabiniomial functions using the 'curve_fit' function of the *scipy.optimize* package. The beta-binomial functions are calculated with the betabinom function of the *scipy.stats* package.


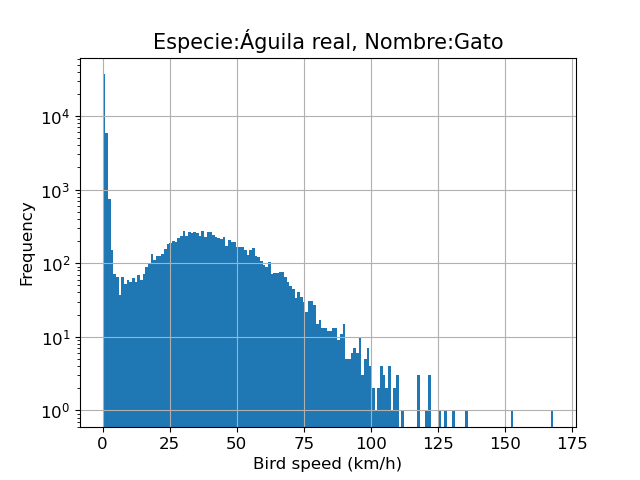


**Figure S1.** Example of flight speed histogram for an individual (i.e., individual “Gato”) of golden eagle.

The optimization process is initialized with the parameters: p0 = [C1=0.80, a1=1.5, b1=1000, C2=0.2, a2=5, b2=15]. Where *C1* refers to the normalizing constant for the first distribution; a1 i.e., alfa value for the first beta-binomial distribution; *b1*: beta value for the first beta-binomial distribution; *C2*: normalizing constant for the second distribution; *a2*: alfa value for the second beta-binomial distribution; *b2*: beta value for the second beta-binomial distribution. Once the histogram has been fitted to this sum of two beta-binomial functions, two groups were defined, those data whose fit is considered satisfactory, and those which were not. The satisfactorily fitted data could be fitted to one of the two beta-binomials, i.e. to the flight one or to non-flying one.


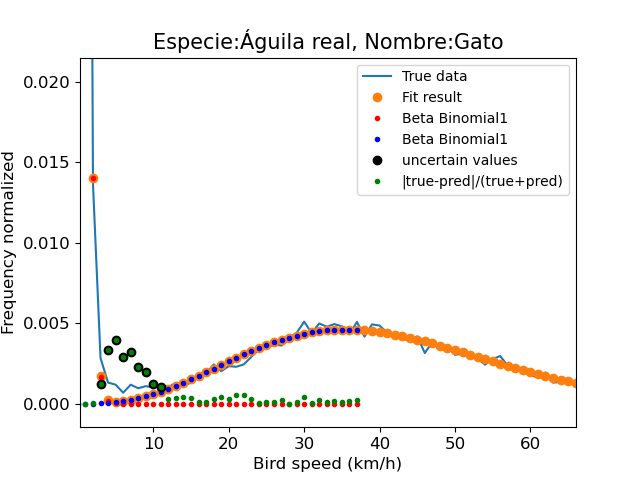


**Figure S2.** Plot where the real data and the fit of the two beta-binomials corresponding to the data of the "non-flying" and "flying" individuals are represented. The red dots are the "no-flying" data fitted to beta-binomial 1 and the blue dots are the “flying” data fitted to beta-binomial 2. The black dots indicate an intermediate zone where the fitting error is triggered.

We consider that a data is satisfactorily adjusted when the difference between the real value and the mean between the adjustment and the real data is < 0.4, that is, if the error in the adjustment is greater than 40% of the mean between the real value and the adjustment, we consider that we do not know if it is flying or not. With the data satisfactorily adjusted we train a machine learning model to which we will give as labels the flight and pose data and the variables 'acc' and 'acc_y'. The accelerometer and magnetometer variables were studied in their three axes separately and in module, i.e. 8 variables. Finally, only the accelerometer module and the accelerometer y-axis were selected because they contained the most information with the minimum number of variables.


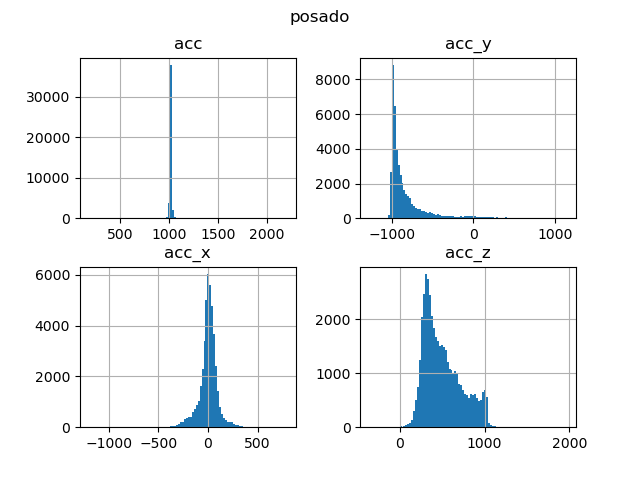


**Figure S3.** Histogram of the frequency of data provided by the accelerometer in modulus and in the x, y, and z axes for the data classified as *non-flying* from the beta-binomial fit.


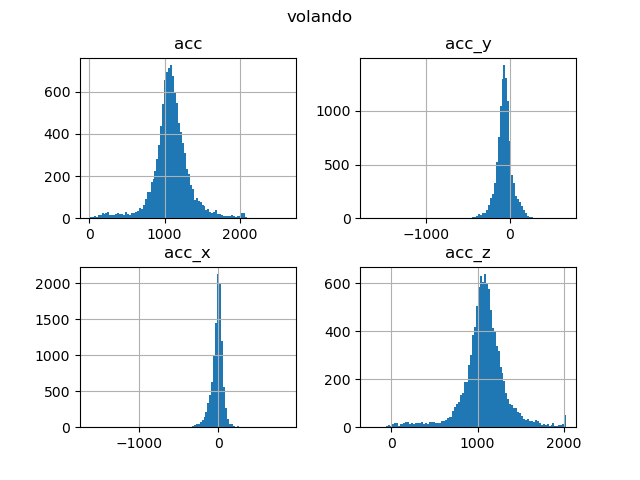


**Figure S4.** Histogram of the frequency of data provided by the accelerometer in modulus and in the x, y, and z axes for the data classified as *flying* from the beta-binomial fit.


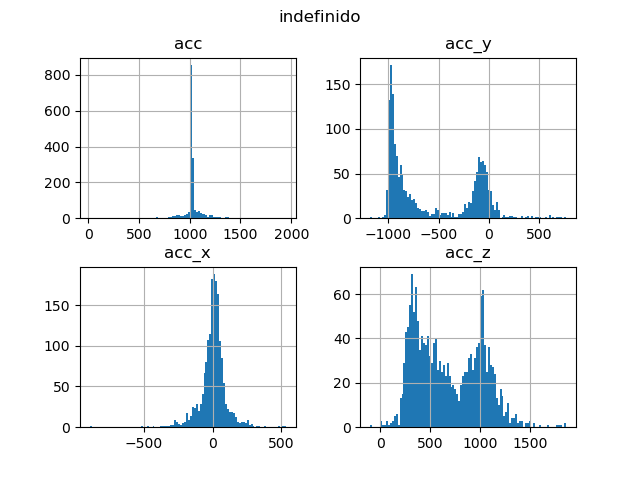


**Figure S5.** Plot of the undefined data where we can observe two clearly differentiated peaks in acc_y that coincide with the peaks in acc_y of the perched and flying data. On the z-axis these two peaks are also observed but with less separation and the correlation with y was high, so it did not contribute new information. Acc provides information regarding the variance of the distribution.

For all individuals we trained our own machine learning model using *kNeigoburs* with 10 neighbors and *minmaxscaler* for prior scaling. It was tested with the training data itself, obtaining in all cases an accuracy higher than 0.97 and kappa higher than 0.9 using *stratiffeid kFold* with 5 splits. Lastly, with the model trained with the data already labeled, we applied the model to the unlabeled data classifying it as perched or flight.

**Table S2.** Exact dates of the different reproductive periods for each species obtained through observational monitoring carried out by filed technicians working in the area.

| **Period** | **Spanish imperial eagle** | **Golden eagle** | **Bonelli's eagle** |
| --- | --- | --- | --- |
| incubation | 15th March - 31st April | 15th March - 31st April | 15th February - 31st April |
| breeding | 1st May - 10th July | 1st May - 10th July | 1st April - 31st May |
| chick dependency | 11st July - 31st August | 11st July - 31st August | 1st June -31st July |
| no breeding period | 1st September - 14th March | 1st September - 14th March | 1st August - 14th February |


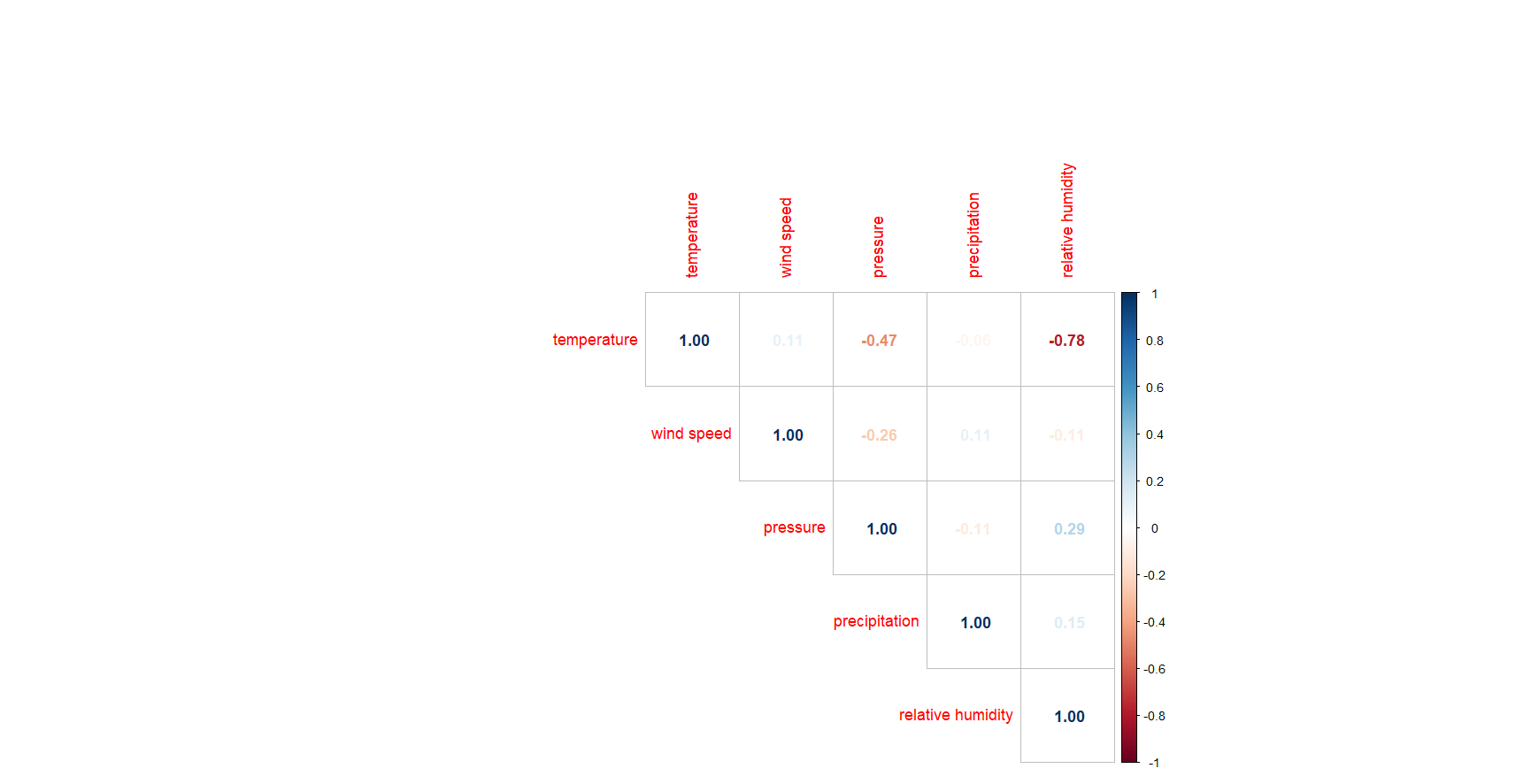


**Figure S6.** Correlation graph showing the values of Pearson's correlation coefficients between different climate variables.

# SECTION S2: Results

| **Model** | **R^2^** | **Factors** | **Estimate** | **SE** | **df** | **t value** | ***p*-value** |  |
| --- | --- | --- | --- | --- | --- | --- | --- | --- |
| temperature*species + hour + breeding season + (1\|individual) | 0.026 | (Intercept) | 3.71 | 0.09 | 29 | 39.88 | 0.00 | *** |
|  |  | **temperature** | **0.14** | **0.01** | **158200** | **17.93** | **0.00** | ******* |
|  |  | species: Golden Eagle | -0.16 | 0.13 | 29 | -1.19 | 0.25 |  |
|  |  | species: Bonelli's Eagle | 0.00 | 0.11 | 29 | -0.02 | 0.98 |  |
|  |  | **hour** | **0.08** | **0.00** | **158300** | **19.04** | **0.00** | ******* |
|  |  | **breeding season: non-breading** | **-0.11** | **0.01** | **155300** | **-13.73** | **0.00** | ******* |
|  |  | temperature * species: Golden Eagle | -0.01 | 0.01 | 157800 | -0.61 | 0.54 |  |
|  |  | **temperature * species: Bonelli's Eagle** | **0.05** | **0.01** | **155900** | **5.23** | **0.00** | ******* |
|  |  |  |  |  |  |  |  |  |
| wind speed*species + hour + breeding season + (1\|individual) | 0.017 | (Intercept) | 3.72 | 0.10 | 28 | 38.77 | 0.00 | *** |
|  |  | wind speed | 0.01 | 0.01 | 158300 | 1.35 | 0.178 |  |
|  |  | species: Golden Eagle | -0.16 | 0.14 | 29 | -1.14 | 0.262 |  |
|  |  | species: Bonelli's Eagle | 0.00 | 0.11 | 29 | -0.03 | 0.975 |  |
|  |  | **hour** | **0.12** | **0.00** | **158200** | **29.70** | **0.00** | ******* |
|  |  | **breeding season: non-breading** | **-0.18** | **0.01** | **155400** | **-23.62** | **0.00** | ******* |
|  |  | **wind speed * species: Golden Eagle** | **0.06** | **0.01** | **158300** | **6.45** | **0.00** | ******* |
|  |  | **wind speed * species: Bonelli's Eagle** | **0.08** | **0.01** | **158300** | **9.02** | **0.00** | ******* |
|  |  |  |  |  |  |  |  |  |
| pressure*species + hour + breeding season + (1\|individual) | 0.015 | (Intercept) | 3.73 | 0.10 | 28 | 39.18 | 0.00 | *** |
|  |  | **pressure** | **0.05** | **0.01** | **158300** | **5.75** | **0.00** | *** |
|  |  | species: Golden Eagle | -0.16 | 0.14 | 29 | -1.15 | 0.26 |  |
|  |  | species: Bonelli's Eagle | -0.01 | 0.11 | 29 | -0.11 | 0.91 |  |
|  |  | **hour** | **0.14** | **0.00** | **158300** | **37.12** | **0.00** | ******* |
|  |  | **breeding season: non-breading** | **-0.20** | **0.01** | **156700** | **-24.13** | **0.00** | ******* |
|  |  | **pressure * species: Golden Eagle** | **-0.04** | **0.01** | **158200** | **-4.28** | **0.00** | ******* |
|  |  | **pressure * species: Bonelli's Eagle** | **-0.07** | **0.01** | **156300** | **-6.94** | **0.00** | ******* |
|  |  |  |  |  |  |  |  |  |
| precipitation*species + hour + breeding season + (1\|individual) | 0.016 | (Intercept) | 3.73 | 0.10 | 28 | 38.24 | 0.00 | *** |
|  |  | **precipitation** | **-0.05** | **0.01** | **158200** | **-8.81** | **0.00** | ******* |
|  |  | species: Golden Eagle | -0.15 | 0.14 | 29 | -1.09 | 0.28 |  |
|  |  | species: Bonelli's Eagle | -0.02 | 0.12 | 29 | -0.19 | 0.85 |  |
|  |  | **hour** | **0.14** | **0.00** | **158200** | **37.81** | **0.00** | ******* |
|  |  | **breeding season: non-breading** | **-0.19** | **0.01** | **155500** | **-24.49** | **0.00** | ******* |
|  |  | precipitation * species: Golden Eagle | 0.01 | 0.01 | 158300 | 1.45 | 0.15 |  |
|  |  | **precipitation * species: Bonelli's Eagle** | **0.03** | **0.01** | **158200** | **3.10** | **0.00** | ****** |

**Table S3.** Model coefficients by means of generalized lineal mixed models (GLMMs) showing the influence of different weather variables (i.e., temperature, wind speed, pressure and precipitation) in raptor *flight height* (m). The estimates of the parameters, the standard error (SE), t value, and signification (p-value) are shown. Significant p-values are in bold. The marginal R^2^ (i.e., variance explained by the fixed effects) is indicated for each model.

**Table S4.** Model coefficients by means of generalized lineal mixed models (GLMMs) showing the influence of different weather variables (i.e., temperature, wind speed, pressure and precipitation) in raptor *flight speed* (km/h). The estimates of the parameters, the standard error (SE), t value, and signification (p-value) are shown. Significant p-values are in bold. The marginal R^2^ (i.e., variance explained by the fixed effects) is indicated for each model.

| **Model** | **R^2^** | **Factors** | **Estimate** | **SE** | **df** | **t value** | ***p*-value** |  |
| --- | --- | --- | --- | --- | --- | --- | --- | --- |
| temperature*species + hour + breeding season + (1\|individual) | 0.032 | (Intercept) | 2.02 | 0.13 | 28 | 16.00 | 0.00 | *** |
|  |  | **temperature** | **0.06** | **0.02** | **120700** | **3.77** | **0.00** | ******* |
|  |  | **species: Golden Eagle** | **-0.83** | **0.18** | **29** | **-4.60** | **0.00** | ******* |
|  |  | **species: Bonelli's Eagle** | **-1.07** | **0.15** | **29** | **-7.13** | **0.00** | ******* |
|  |  | **hour** | **0.04** | **0.01** | **120700** | **5.48** | **0.00** | ******* |
|  |  | **breeding season: non-breading** | **-0.06** | **0.02** | **114700** | **-3.87** | **0.00** | ******* |
|  |  | **temperature * species: Golden Eagle** | **-0.08** | **0.02** | **119600** | **-4.14** | **0.00** | ******* |
|  |  | **temperature * species: Bonelli's Eagle** | **0.15** | **0.02** | **117300** | **8.15** | **0.00** | ******* |
|  |  |  |  |  |  |  |  |  |
| wind speed*species + hour + breeding season + (1\|individual) | 0.34 | (Intercept) | 2.01 | 0.12 | 29 | 16.20 | 0.00 | *** |
|  |  | wind speed | 0.02 | 0.01 | 120800 | 1.71 | 0.0882 | . |
|  |  | **species: Golden Eagle** | **-0.82** | **0.18** | **29** | **-4.63** | **0.00** | ******* |
|  |  | **species: Bonelli's Eagle** | **-1.07** | **0.15** | **29** | **-7.31** | **0.00** | ******* |
|  |  | **hour** | **0.03** | **0.01** | **120800** | **4.36** | **0.00** | ******* |
|  |  | **breeding season: non-breading** | **-0.05** | **0.01** | **114100** | **-3.24** | **0.00** | ****** |
|  |  | **wind speed * species: Golden Eagle** | **0.16** | **0.02** | **120800** | **8.12** | **0.00** | ******* |
|  |  | **wind speed * species: Bonelli's Eagle** | **0.17** | **0.02** | **120800** | **10.33** | **0.00** | ******* |
|  |  |  |  |  |  |  |  |  |
| pressure*species + hour + breeding season + (1\|individual) | 0.032 | (Intercept) | 2.04 | 0.12 | 28 | 16.34 | 0.00 | *** |
|  |  | **pressure** | **0.14** | **0.02** | **120800** | **9.07** | **0.00** | ******* |
|  |  | **species: Golden Eagle** | **-0.82** | **0.18** | **29** | **-4.61** | **0.00** | ******* |
|  |  | **species: Bonelli's Eagle** | **-1.09** | **0.15** | **29** | **-7.37** | **0.00** | ******* |
|  |  | **hour** | **0.10** | **0.01** | **120800** | **13.68** | **0.00** | ******* |
|  |  | **breeding season: non-breading** | **-0.13** | **0.02** | **117200** | **-8.24** | **0.00** | ******* |
|  |  | pressure * species: Golden Eagle | 0.00 | 0.02 | 120700 | 0.24 | 0.81 |  |
|  |  | **pressure * species: Bonelli's Eagle** | **-0.16** | **0.02** | **117900** | **-8.53** | **0.00** | ******* |
|  |  |  |  |  |  |  |  |  |
| precipitation*species + hour + breeding season + (1\|individual) | 0.031 | (Intercept) | 2.02 | 0.13 | 28 | 15.88 | 0.00 | *** |
|  |  | **precipitation** | **-0.04** | **0.01** | **120800** | **-3.74** | **0.00** | ******* |
|  |  | **species: Golden Eagle** | **-0.81** | **0.18** | **29** | **-4.48** | **0.00** | ******* |
|  |  | **species: Bonelli's Eagle** | **-1.11** | **0.15** | **29** | **-7.37** | **0.00** | ******* |
|  |  | **hour** | **0.09** | **0.01** | **120800** | **12.62** | **0.00** | ******* |
|  |  | **breeding season: non-breading** | **-0.06** | **0.01** | **114400** | **-4.48** | **0.00** | ******* |
|  |  | precipitation * species: Golden Eagle | 0.00 | 0.02 | 120800 | 0.06 | 0.95 |  |
|  |  | precipitation * species: Bonelli's Eagle | 0.02 | 0.02 | 120800 | 1.39 | 0.16 |  |

**Table S5.** Model coefficients by means of generalized lineal mixed models (GLMMs) showing the influence of different weather variables (i.e., temperature, wind speed, pressure and precipitation) in raptor *distance traveled per hour* (km). The estimates of the parameters, the standard error (SE), t value, and signification (p-value) are shown. Significant p-values are in bold. The marginal R^2^ (i.e., variance explained by the fixed effects) is indicated for each model.

| **Model** | **R^2^** | **Factors** | **Estimate** | **SE** | **df** | **t value** | ***p*-value** |  |
| --- | --- | --- | --- | --- | --- | --- | --- | --- |
| temperature*species + hour + breeding season + (1\|individual) | 0.011 | (Intercept) | 0.68 | 0.16 | 29 | 4.37 | 0.00 | *** |
|  |  | temperature | 0.02 | 0.01 | 163400 | 1.65 | 0.10 | . |
|  |  | species: Golden Eagle | -0.23 | 0.22 | 29 | -1.03 | 0.31 |  |
|  |  | **species: Bonelli's Eagle** | **-0.38** | **0.18** | **29** | **-2.08** | **0.05** | ***** |
|  |  | **hour** | **0.08** | **0.01** | **163400** | **16.32** | **0.00** | ******* |
|  |  | **breeding season: non-breading** | **-0.07** | **0.01** | **162400** | **-6.87** | **0.00** | ******* |
|  |  | temperature * species: Golden Eagle | -0.01 | 0.01 | 163300 | -0.93 | 0.35 |  |
|  |  | **temperature * species: Bonelli's Eagle** | **0.11** | **0.01** | **162600** | **8.78** | **0.00** | ******* |
|  |  |  |  |  |  |  |  |  |
| wind speed*species + hour + breeding season + (1\|individual) | 0.019 | (Intercept) | 0.66 | 0.16 | 29 | 4.14 | 0.00 | *** |
|  |  | **wind speed** | **0.09** | **0.01** | **163400** | **9.81** | **0.00** | ******* |
|  |  | species: Golden Eagle | -0.21 | 0.22 | 29 | -0.92 | 0.36 |  |
|  |  | species: Bonelli's Eagle | -0.35 | 0.19 | 29 | -1.87 | 0.07 | . |
|  |  | **hour** | **0.04** | **0.00** | **163400** | **8.82** | **0.00** | ******* |
|  |  | **breeding season: non-breading** | **-0.06** | **0.01** | **162500** | **-5.94** | **0.00** | ******* |
|  |  | **wind speed * species: Golden Eagle** | **0.16** | **0.01** | **163400** | **12.56** | **0.00** | ******* |
|  |  | **wind speed * species: Bonelli's Eagle** | **0.13** | **0.01** | **163400** | **11.58** | **0.00** | ******* |
|  |  |  |  |  |  |  |  |  |
| pressure*species + hour + breeding season + (1\|individual) | 0.012 | (Intercept) | 0.71 | 0.16 | 29 | 4.45 | 0.00 | *** |
|  |  | **pressure** | **0.15** | **0.01** | **163400** | **14.65** | **0.00** | ******* |
|  |  | species: Golden Eagle | -0.23 | 0.22 | 29 | -1.02 | 0.32 |  |
|  |  | **species: Bonelli's Eagle** | **-0.40** | **0.19** | **29** | **-2.12** | **0.04** | ***** |
|  |  | **hour** | **0.12** | **0.00** | **163400** | **24.69** | **0.00** | ******* |
|  |  | **breeding season: non-breading** | **-0.14** | **0.01** | **162900** | **-13.05** | **0.00** | ******* |
|  |  | **pressure * species: Golden Eagle** | **-0.05** | **0.01** | **163400** | **-3.96** | **0.00** | ******* |
|  |  | **pressure * species: Bonelli's Eagle** | **-0.15** | **0.01** | **162800** | **-12.01** | **0.00** | ******* |
|  |  |  |  |  |  |  |  |  |
| precipitation*species + hour + breeding season + (1\|individual) | 0.011 | (Intercept) | 0.68 | 0.16 | 29 | 4.21 | 0.00 | *** |
|  |  | **precipitation** | **-0.06** | **0.01** | **163400** | **-7.72** | **0.00** | ******* |
|  |  | species: Golden Eagle | -0.22 | 0.23 | 29 | -0.96 | 0.35 |  |
|  |  | **species: Bonelli's Eagle** | **-0.41** | **0.19** | **29** | **-2.14** | **0.04** | ***** |
|  |  | **hour** | **0.11** | **0.00** | **163400** | **23.17** | **0.00** | ******* |
|  |  | **breeding season: non-breading** | **-0.08** | **0.01** | **162500** | **-7.84** | **0.00** | ******* |
|  |  | precipitation * species: Golden Eagle | 0.02 | 0.01 | 163400 | 1.71 | 0.09 | . |
|  |  | **precipitation * species: Bonelli's Eagle** | **0.05** | **0.01** | **163400** | **4.63** | **0.00** | ******* |

**Table S6.** Model coefficients by means of generalized lineal mixed models (GLMMs) showing the influence of different weather variables (i.e., temperature, wind speed, pressure and precipitation) in raptor *proportion of time flown per hour*. The estimates of the parameters, the standard error (SE), t value, and signification (p-value) are shown. Significant p-values are in bold. The marginal R^2^ (i.e., variance explained by the fixed effects) is indicated for each model.

| **Model** | **R^2^** | **Factors** | **Estimate** | **SE** | **t value** | **p-value** |  |
| --- | --- | --- | --- | --- | --- | --- | --- |
| temperature*species + hour + breeding season + (1\|individual) | 0.006 | (Intercept) | 0.91 | 0.06 | 15.10 | 0.00 | *** |
|  |  | **temperature** | **0.04** | **0.01** | **3.60** | **0.00** | ******* |
|  |  | **species: Golden Eagle** | **-0.18** | **0.09** | **-2.13** | **0.03** | ***** |
|  |  | species: Bonelli's Eagle | 0.03 | 0.07 | 0.38 | 0.70 |  |
|  |  | **hour** | **-0.02** | **0.01** | **-3.76** | **0.00** | ******* |
|  |  | **breeding season: non-breading** | **0.07** | **0.01** | **5.69** | **0.00** | ******* |
|  |  | **temperature * species: Golden Eagle** | **-0.07** | **0.02** | **-4.76** | **0.00** | ******* |
|  |  | **temperature * species: Bonelli's Eagle** | **-0.17** | **0.01** | **-11.85** | **0.00** | ******* |
|  |  |  |  |  |  |  |  |
| wind speed*species + hour + breeding season + (1\|individual) | 0.01 | (Intercept) | 0.93 | 0.06 | 15.26 | 0.00 | *** |
|  |  | **wind speed** | **-0.09** | **0.01** | **-8.48** | **0.00** | ******* |
|  |  | **species: Golden Eagle** | **-0.19** | **0.09** | **-2.22** | **0.03** | ***** |
|  |  | species: Bonelli's Eagle | 0.00 | 0.07 | 0.05 | 0.96 |  |
|  |  | hour | 0.00 | 0.01 | 0.79 | 0.43 |  |
|  |  | **breeding season: non-breading** | **0.05** | **0.01** | **4.72** | **0.00** | ******* |
|  |  | **wind speed * species: Golden Eagle** | **-0.07** | **0.01** | **-4.59** | **0.00** | ******* |
|  |  | **wind speed * species: Bonelli's Eagle** | **-0.09** | **0.01** | **-6.65** | **0.00** | ******* |
|  |  |  |  |  |  |  |  |
| pressure*species + hour + breeding season + (1\|individual) | 0.004 | (Intercept) | 0.89 | 0.06 | 14.42 | 0.00 | *** |
|  |  | **pressure** | **-0.08** | **0.01** | **-7.02** | **0.00** | ******* |
|  |  | **species: Golden Eagle** | **-0.18** | **0.09** | **-1.98** | **0.05** | ***** |
|  |  | species: Bonelli's Eagle | 0.04 | 0.07 | 0.51 | 0.61 |  |
|  |  | **hour** | **-0.05** | **0.01** | **-8.75** | **0.00** | ******* |
|  |  | **breeding season: non-breading** | **0.10** | **0.01** | **7.93** | **0.00** | ******* |
|  |  | **pressure * species: Golden Eagle** | **0.03** | **0.02** | **2.28** | **0.02** | ***** |
|  |  | **pressure * species: Bonelli's Eagle** | **0.10** | **0.01** | **7.17** | **0.00** | ******* |
|  |  |  |  |  |  |  |  |
| precipitation*species + hour + breeding season + (1\|individual) | 0.004 | (Intercept) | 0.90 | 0.06 | 14.09 | 0.00 | *** |
|  |  | precipitation | 0.01 | 0.01 | 1.02 | 0.31 |  |
|  |  | species: Golden Eagle | -0.18 | 0.09 | -1.95 | 0.05 | . |
|  |  | species: Bonelli's Eagle | 0.05 | 0.08 | 0.65 | 0.52 |  |
|  |  | **hour** | **-0.04** | **0.01** | **-8.26** | **0.00** | ******* |
|  |  | **breeding season: non-breading** | **0.07** | **0.01** | **5.99** | **0.00** | ******* |
|  |  | precipitation * species: Golden Eagle | 0.01 | 0.02 | 0.59 | 0.56 |  |
|  |  | precipitation * species: Bonelli's Eagle | -0.01 | 0.01 | -0.43 | 0.67 |  |

**Table S7.** Model coefficients by means of generalized lineal mixed models (GLMMs) showing the influence of heatwaves in flight height and speed, the distance traveled per hour and the proportion of time flown per hour, and the differential influence among species (i.e., interaction between *species* and *heatwave*). The estimates of the parameters, the standard error (SE), t value, and signification (*p*-value) are shown. Significant *p*-values are in bold. The marginal R^2^ (i.e., variance explained by the fixed effects) is indicated for each model.

| **Response variable** | **R^2^** | **Factors** | **Estimate** | **SE** | **t value** | ***p*-value** | |
| --- | --- | --- | --- | --- | --- | --- | --- |
| Flight height | 0.015 | (Intercept) | 3.75 | 0.10 | 39.06 | 0.00 | *** |
|  |  | **heatwave: heatwave** | **-0.12** | **0.03** | **-4.90** | **0.00** | ******* |
|  |  | species: Golden Eagle | -0.15 | 0.14 | -1.13 | 0.27 |  |
|  |  | species: Bonelli's Eagle | -0.03 | 0.11 | -0.28 | 0.78 |  |
|  |  | **hour** | **0.14** | **0.00** | **37.62** | **0.00** | ******* |
|  |  | **breeding season: non-breading** | **-0.20** | **0.01** | **-25.81** | **0.00** | ******* |
|  |  | heatwave: heatwave * species: Golden Eagle | -0.02 | 0.04 | -0.63 | 0.53 |  |
|  |  | **heatwave: heatwave * species: Bonelli's Eagle** | **0.12** | **0.03** | **4.03** | **0.00** | ******* |
|  |  |  |  |  |  |  |  |
| Flight speed | 0.032 | (Intercept) | 2.07 | 0.12 | 16.73 | 0.00 | *** |
|  |  | **heatwave: heatwave** | **-0.31** | **0.05** | **-6.22** | **0.00** | ******* |
|  |  | **species: Golden Eagle** | **-0.79** | **0.18** | **-4.48** | **0.00** | ******* |
|  |  | **species: Bonelli's Eagle** | **-1.13** | **0.15** | **-7.71** | **0.00** | ******* |
|  |  | **hour** | **0.09** | **0.01** | **12.72** | **0.00** | ******* |
|  |  | **breeding season: non-breading** | **-0.10** | **0.01** | **-7.10** | **0.00** | ******* |
|  |  | **heatwave: heatwave * species: Golden Eagle** | **-0.23** | **0.07** | **-3.33** | **0.00** | ******* |
|  |  | **heatwave: heatwave * species: Bonelli's Eagle** | **0.23** | **0.06** | **3.93** | **0.00** | ******* |
|  |  |  |  |  |  |  |  |
| Distance traveled per hour | 0.013 | (Intercept) | 0.74 | 0.16 | 4.66 | 0.00 | *** |
|  |  | **heatwave: heatwave** | **-0.36** | **0.03** | **-11.43** | **0.00** | ******* |
|  |  | species: Golden Eagle | -0.21 | 0.23 | -0.95 | 0.35 |  |
|  |  | **species: Bonelli's Eagle** | **-0.42** | **0.19** | **-2.23** | **0.03** | ***** |
|  |  | **hour** | **0.11** | **0.00** | **23.23** | **0.00** | ******* |
|  |  | **breeding season: non-breading** | **-0.12** | **0.01** | **-11.97** | **0.00** | ******* |
|  |  | heatwave: heatwave * species: Golden Eagle | -0.08 | 0.04 | -1.81 | 0.07 | . |
|  |  | **heatwave: heatwave * species: Bonelli's Eagle** | **0.13** | **0.04** | **3.41** | **0.00** | ******* |
|  |  |  |  |  |  |  |  |
| Proportion of time flown per hour | 0.005 | (Intercept) | 0.88 | 0.06 | 14.10 | 0.00 | *** |
|  |  | **heatwave: heatwave** | **0.16** | **0.04** | **4.13** | **0.00** | ******* |
|  |  | **species: Golden Eagle** | **-0.19** | **0.09** | **-2.13** | **0.03** | ***** |
|  |  | species: Bonelli's Eagle | 0.05 | 0.07 | 0.70 | 0.49 |  |
|  |  | **hour** | **-0.05** | **0.01** | **-8.31** | **0.00** | ******* |
|  |  | **breeding season: non-breading** | **0.09** | **0.01** | **7.74** | **0.00** | ******* |
|  |  | **heatwave: heatwave * species: Golden Eagle** | **0.11** | **0.05** | **2.08** | **0.04** | ***** |
|  |  | heatwave: heatwave * species: Bonelli's Eagle | -0.05 | 0.05 | -1.10 | 0.27 |  |
